# Focal Segmental Glomerulosclerosis in Paediatric Population of South Punjab Pakistan: A Tertiary Care Hospital Experience

**DOI:** 10.12669/pjms.37.2.3535

**Published:** 2021

**Authors:** Rabia Saleem Safdar, M. Faisal Mehar, Afsheen Asghar Khan, Nusrat Buzdar

**Affiliations:** 1 Rabia Saleem Safdar, FCPS (Pediatric Medicine), Department of Pediatrics, Ward Number 19, Nishtar Medical University Hospital, Multan, Pakistan; 2 M. Faisal Mehar, FCPS (Pediatric Medicine), Department of Pediatrics, Ward Number 19, Nishtar Medical University Hospital, Multan, Pakistan; 3 Afsheen Asghar, FCPS (Pediatric Medicine), Department of Pediatrics, Ward Number 19, Nishtar Medical University Hospital, Multan, Pakistan; 4 Nusrat Buzdar, FCPS (Pediatric Medicine), Department of Pediatrics, Ward Number 19, Nishtar Medical University Hospital, Multan, Pakistan

**Keywords:** Focal segmental glomerulosclerosis, Complete remission, End stage kidney disease, Immunosuppressive drugs

## Abstract

**Objectives:**

To find out frequency, clinicopathological features, response of treatment and outcome among children with primary focal segmental glomerulosclerosis (FSGS).

**Methods:**

This retrospective, non-interventional medical charts review study was conducted from a period of January 2011 to January 2020 at Pediatric Department of Nishtar Medical University Hospital, Multan, Pakistan. During the nine years study period, children of both genders, aged less than 16 years, with renal biopsies proven FSGS were included. Patient’s demographic along with clinical and laboratory data, urine dipstick for proteinuria, renal functions, 24 hours urinary protein and ultrasonography findings of kidneys, ureters and bladder (KUB) were noted from case records. Response rates of various treatment options and their outcome like remission, partial remission, no remission with stable kidney disease & no remission with progression of kidney disease were noted.

**Results:**

During the study duration, out of 307 renal biopsies performed in glomerulonephritis cases, 124 (40.4%) had primary FSGS. In 124 primary FSGS cases, mean age was 8.83±3.05 years while most of the children, 70 (56.5%) were above 10 years of age. Majority of the cases, 64 (51.6%) were male. Mean follow up duration was noted to be 28.35+18.47 months. Most of the cases, 68 (54.8%) were found to have complete remission, 22 (17.7%) partial remission while 11 (8.9%) progressed to ESKD.

**Conclusions:**

Among children, frequency of primary FSGS was high at our setting. Most of the cases achieved sustained remission rates with the help of immunosuppressive drugs. Cyclosporine and tacrolimus were found to be the most effective drugs.

## INTRODUCTION

Focal segmental glomerulosclerosis (FSGS) is characterized by steroid resistant nephrotic syndrome (SRNS) as well as segmental sclerosis that involve some of the glomeruli.^1^ Among pediatric age groups, FSGS is estimated to account for around 7 to 20% of all forms of idiopathic nephrotic syndrome (NS).^2^,^3^ FSGS is also noted to be commonly associated with hematuria and hypertension. At earlier stages, FSGS is difficult to distinguish from minimal change disease (MCD).^4^ Globally, incidence of FSGS is on the rise.^5^ Canada reports 0.4 to 0.9 new cases per 100000 children annually.^6^ Local data from Pakistan showed FSGS to be the commonest histopathological pattern among children having SRNS.^7^

FSGS is considered to have variable outcomes as half of the cases are observed to reach end stage kidney disease (ESKD) when they reach 15 years of age whereas multi-centric data from developing countries noted FSGS to be the commonest form of GS contributing to 10.2% pediatric cases that ended up as ESKD.^8^,^9^

Natural history of FSGS has been elaborated but its management is still a topic of concern. FSGS is the most common form of SRNS among children in Pakistan but scarcity of details exists at local level about its clinicopathological analysis and outcome. This is the 1^st^ retrospective analysis of pediatric age group having biopsy proven FSGS from South Punjab Pakistan. This study was aimed to find out frequency, clinicopathological features, response of treatment and outcome among children with primary FSGS.

## METHODS

This retrospective, non-interventional medical charts review study was conducted from a period of January 2011 to January 2020 at Pediatric Department of Nishtar Medical University Hospital, Multan, Pakistan. During this nine years period, a total of 307 children of both gender, aged less than 16 years, with renal biopsies proven FSGS with the help of light microscopy (LM) and immunofluorescence (IF) were included. Out of those 307 cases, those having FSGS, with more than six months of follow up were further analyzed in this study. All children having secondary etiologies of FSGS or those who lost follow up were not included in the final analysis. Primary FSGS were the cases who are proved on biopsy as FSGS without underlying pathology. Secondary cases were those who had underlying pathology like HIV, Hepatitis, Sickle cell disease, Hemolytic ureamic syndrome etc and were excluded by clinical evaluation and laboratory evaluation. Lost to follow up were those children who lost visit for three months at any time during the study period of nine year before categorization. Approval from Institutional Ethical Committee (Ref. No. 15226, Dated: 12-08-2020) was taken for this study.

Patient’s demographic along with clinical and laboratory data, urine dipstick for proteinuria, renal functions, 24 hours urinary protein and ultrasonography findings of kidneys, ureters and bladder (KUB) were noted from case records. Disease definitions as well treatment outcomes were labeled as per “The Kidney Disease: Improving Global Outcomes” (KDIGO) 2012 guidelines. Outcome like remission, partial remission, no remission with stable kidney disease & no remission with progression of kidney disease were followed for the period of 12 months, if remained constant during the said period, were finally considered the final outcome and added in data. Outcome as ESKD was also noted. Complete Remission was labeled as proteinuria <4 mg/m2/hour or nil to trace on urine dipstick testing for three consecutive days. Partial Remission was taken as proteinuria >4 mg/ m2 /hour but < 40 mg/ m2 /hour or 1+ to 2+ protein on urine dipstick testing for 3 consecutive days. Relapse was labeled as proteinuria >40 mg /m2 /hour or protein: creatinine ratio >2 or ≥3+protein on urine dipstick testing for three consecutive days. No remission with stable kidney disease was considered as proteinuria >40 mg /m2 /hour or protein: creatinine ratio >2 or ≥3+ protein on urine dipstick testing for three consecutive days along with normal renal parameters. No remission with progression of kidney disease was proteinuria >40 mg /m2 /hour or protein: creatinine ratio >2 or ≥3+ protein on urine dipstick testing for 3 consecutive days along with deranged renal parameters. End stage kidney disease was marked as chronic kidney disease with stage v (GFR <15 ml/minute/1.73m2).

Institutional standard treatment protocols as per MENDOZA treatment were used in the initial years of study. Prednisolone 60mg/m2/day for duration of 4-6 weeks as standard treatment. If remission noted, dosage was reduced as 40mg/m2/day every alternate day for further duration of 4 weeks and gradual tapering over three to six months. Pulse methyl-prednisolone was started after taking biopsy of those who did not respond to steroids over eight weeks duration according to MENDOZA protocol and cyclophosphamide was also added in steroid resistant cases. Those who didn’t respond to MENDOZA protocol, were started cyclosporine and sustained remission. In last five years, 2nd line immunosuppressive drugs were used as center protocol for steroid resistant cases of FSGS. Cyclosporine was the commonest 2nd line choice as three to five mg/kg/day in two divided dosages while keeping a target between 100-150 ng/dL in mind for a period of 24 months in cases which responded or stopped in cases that had failure in showing partial or complete response. Tacrolimus (0.1mg/kg/day in 2 divided dosages) and mycophenloate mofetil (1200mg/m2/day in 2 divided dosages) were some of the other 2nd line treatments in the later years of this study. Tapering off of drugs was done as per clinical response. SPSS (Statistical Package for Social Science USA) version 24.0 was used for data entry and analysis. Frequency and percentages were of qualitative variables were described as percentages and frequencies while quantitative variables were highlighted as mean and standard deviation.

## RESULTS

During the study duration, out of 307 renal biopsies performed in GN cases, 124 (40.4%) cases were noted to have FSGS. These 124 cases were considered for further analysis. In 124 FSGS cases, mean age was 8.83±3.05 years while most of the children, 70 (56.5%) were above 10 years of age. Majority of the cases, 64 (51.6%) were male representing a male to female ratio of 1.07:1. (Table-I)

**Table-I:** Characteristics of the Patients.

*Total Cases having FSGS*	*124*
Gender	
Male	64 (51.6%)
Female	60 (48.4%)
Age (Mean±SD)	8.83±3.05
***Age Groups (years)***	
≤10	54 (43.5%)
>10	70 (56.5%)

At the time of admission, microscopic hematuria was noted among 28 (22.6%) cases. Mean red blood cell count per microliter was noted as 109.35+108 on urinary dipstick test. Mean serum albumin was noted as 1.83±0.83 gm/dL while mean serum creatinine was recorded as 0.52±0.35 mg/100mL. There were 48 (38.7%) cases with hypertensive. IF findings showed that 13 (10.5%) cases were IgG positive, 24 (19.4%) IgM positive, 4 (3.2%) C3 positive while 83 (66.9%) had negative IF findings.

In the 1^st^ four year of study period, standard treatment as intravenous methyl-prednisolone was started in all 30 (24.2%) cases, out of which, 12 (40.0%) got complete remission and eight (40.0%) got partial remission. Cyclophosphamide along with methyl-prednisolone pulse was used in all those cases who did not respond to methyl-prednisolone therapy, out of which, 16 (53.3%) reported relapse within two years of stopping this protocol and they were started on cyclosporine and achieved sustained remission. Those who showed cyclosporine toxicity or intolerance, were started on tacrolimum or MMF. Tacrolimus is not cost effective so was not the choice in all cases. The response rates of different drugs used is shown in table number two. There were 4 cases of familial FSGS in our cases and they were started on supportive treatment which was diuretics and angiotensin converting enzyme (ACE) inhibitors. In these familial FSGS cases, three developed ESKD out of which one expired while one remaining cases developed progressive kidney disease. The response rates of different drugs used is shown in Table-II.

**Table-II:** Response of Various Drugs Used

*Drugs*	*No. (%)*
Methyl-prednisolone pulse with Cyclophosphamide	30
Complete Remission	12 (40.0%)
Partial Remission	8 (26.7%)
No-remission with Stable Kidney Disease	5 (16.7%)
No-remission with Progressive Kidney Disease	3 (10.0%)
ESKD	2 (6.7%)
Cyclosporine	65
Complete Remission	43 (66.2%)
Partial Remission	10 (15.4%)
No-remission with Stable Kidney Disease	5 (7.7%)
No-remission with Progressive Kidney Disease	2 (3.1%)
ESKD	5 (7.7%)
Tacrolimus	13
Complete Remission	9 (69.2%)
Partial Remission	1 (7.7%)
No-remission with Stable Kidney Disease	1 (7.7%)
No-remission with Progressive Kidney Disease	1 (15.4%)
ESKD	1 (7.7%)
Mycophenloate Mofetil	12
Complete Remission	4 (33.3%)
Partial Remission	3 (25.0%)
No-remission with Stable Kidney Disease	3 (25.0%)
No-remission with Progressive Kidney Disease	1 (8.3%)
ESKD	1 (8.3%)

Mean follow up duration was noted to be 28.35+18.47 months. The outcome of studied cases is shown in Table-III. Most of the cases, 68 (54.8%) were found to have complete remission, 22 (17.7%) partial remission while 11 (8.9%) progressed to ESKD (1 case expired, two referred to Sindh Institute of Urology and Transplantation [SUIT], Karachi for kidney transplant while eight are on dialysis at our follow up). All those cases who reported complete remission are having sustained remission up until the end of the study period.

**Table-III:** Outcome at the End of the Study Period.

*Outcome*	*Number (%)*
Complete Remission	68 (54.8%)
Partial Remission	22 (17.7%)
No-remission with Stable Kidney Disease	14 (11.3%)
No-remission with Progressive Kidney Disease	9 (5.6%)
End Stage Kidney Disease	11 (8.9%)

## DISCUSSION

During nine years of study period, among renal biopsies performed in GN cases, 40.4% were noted to have FSGS. Spectrum of GN has been found varying across different parts of the world. From Croatia, Batanic et al.^10^ noted very similar findings where they noted FSGS to be among 24.6% GN cases followed by Mesangioproliferative glomerulonephritis (MesPGN) in 19.2%. A study from Bangladesh^11^ found MesPGN to be the commonest histological finding among children with GN. From Pakistan, MCD followed by FSGS has been found to be the most common histopathological findings in GN in a previous research.^12^ These variations could be because of differences subjected to biopsy indications as well as patient referrals and racial predispositions among different nephropathies.^13^,^14^ No standard guideline exists regarding kidney biopsies among pediatric population.

Although clinicopathological aspects of FSGS have been well elaborated in the last few decades since its 1^st^ description by Rich in 1957, management of FSGS has always been challenging and a topic that has been debated over the years.^14^,^15^ Corticosteroids have been the mainstay of FSGS treatment while response of steroid therapy is considered to be the finest predictor of long-term outcome and prognosis. In the study, initially, steroids were used to treat FSGS children. Cyclophosphamide was added later those cases which did not respond to steroid treatment alone. It has been well established from the past findings that long term usage of steroids among SDNS children is associated with increased exposure to steroid related toxicity like osteoporosis, growth disorders, cataract, diabetes mellitus as well as psycho-emotional disorders.^14^,^16^ Various immunosuppressive drugs are supplemented with steroids commonly to avoid steroid toxicity. Our approach was quite similar to the one that has been described previously.^14^,^17^ In the current study, 40% of the children achieved complete remission and 26.7% achieved partial remission with the administration of methyl-prednisolone pulse along with cyclophosphamide. International literature showed complete remission rate of cyclophosphamide to be 51% while partial remission noted in 23% and no response in 26%.^18^ Higher rates of complete remission as 69.4% have been reported in the past by local researcher with cyclophosphamide.^14^ Complete remission among 33.3% children was noted by Brazilian researchers using a combination of steroid and cyclophosphamide.^19^ It was also noted in the present study that 53.3% of the cases using this combination therapy reported relapse within two years of stopping this protocol and they were started on cyclosporine and achieved sustained remission. Previous local data also found 36% of the patients using cyclophosphamide and steroid to report relapse later and were shifted to cyclosporine who achieved sustained remission.^14^

In FSGS, cyclosporine has been seen to minimize the relapse rates by 80%.^20^ In the present study, cyclosporine was found to be the most effective drug, achieving complete remission in 66.2% cases while 15.4% had partial remission and 7.7% were reported with ESKD. Previously published local study found complete remission as 52.6% cases while no response was seen in 12.2%.^14^ Researchers from other parts of the world^21^ have also noted cyclosporine to produce complete remission rates of 44.4% while data from the US found it much higher as 87% among SRNS patients.^22^ Difference in standardization as well as duration could be the one major reason for this variability in response with cyclosporine in children with FSGS. Dependency upon cyclosporine is another aspect that needs to be considered as is the cases with steroid therapy.

Tacrolimus was administered in 13 cases which were resistant to cyclosporine and complete remission was noted in 69.2% of these cases. Recently, tacrolimus has been recommended as an alternative choice to cyclosporine for SRNS and has been shown to be well tolerated showing a complete remission rate of as high as 81%.^15^,^22^,^23^ MFM was used instead of tacrolimus in 12 cases while complete remission among 33.3% and partial remission in 25% cases were reported which is quite similar to previously published studies.^22^,^24^

In the present study 5.6% cases had progression in their kidney disease and 8.9% ended up in ESKD. Worldwide literature present wide variability of 13-78% cases ending up as ESKD in a follow up period of as long as 20 years which again emphasizes the variability in treatment approaches in children having FSGS.^7^,^14^,^16^

### Strength and Limitations of the study

Uniformity in the diagnostic and histopathological approach as good sample size and long duration of follow up are some of the strengths of this study. Selection bias among the study participants could be a limitation, as is usually the case among biopsy base researchers. We also could not treat all the cases as per a single standard protocol over the years which can also present difference in outcomes regarding different therapeutic options. Comparatively shorter duration of follow up is another limitation of this study.

## CONCLUSIONS

Among children, frequency of primary FSGS was high at our setting. Most of the cases achieved sustained remission rates with the help of immunosuppressive drugs. Cyclosporine and tacrolimus were found to be the most effective drugs.

### Authors’ Contribution:

**RSS:** Data Analysis, Drafting, responsible for data’s authenticity and integrity.

**MFM:** Literature Review, Introduction.

**AA:** Data Interpretation, Proof Reading.

**NB:** Literature Review, Discussion.
